# Gender differences in skilled performance under failure competitive environments: evidence from elite archers

**DOI:** 10.3389/fpsyg.2024.1468978

**Published:** 2024-09-23

**Authors:** Chunhua Li, Yangqing Zhao

**Affiliations:** School of Physical Education and Health, Wenzhou University, Wenzhou, Zhejiang, China

**Keywords:** performance under pressure, gender differences, cold hand, feedback, competition

## Abstract

**Introduction:**

Psychologists are particularly interested in how people operate in stressful settings. The sporting arena is a “natural laboratory” for studying how people behave and perform in high-pressure situations. This study explores the gender differences in archers’ ability to cope with adversity, highlighting the significant cold-hand effect observed in both male and female archers, with notable differences in the last arrow performance under pressure.

**Methods:**

Our method is a Poisson general linear model -based test for the cold hand that examines how the performance of the last arrow per set depends on the performance of the previous two shots. We also interact the player’s gender with performance on the previous two arrows and game status to test for gender differences in response to past performance and intermediate game status.

**Results:**

The Poisson regression analysis reveals that male and female archers’ performance dropped significantly after experiencing two consecutive missing bullseyes, which means a cold-hand effect exists. However, although there was no significant difference in the performance of male and female archers on the third arrow, female archers have significantly lower last arrow per set scores than male archers after near poor performance or being in a situation where losing can only be avoided by winning the current set.

**Discussion:**

This finding suggests that female archers are more vulnerable to the potentially negative effects of adversity caused by trailing or recent failures than their male counterparts. We attempt to explain the reasons behind the results above from both psychological and physiological perspectives.

## 1 Introduction

Psychologists are particularly interested in how people operate in stressful settings. The sporting arena is a “natural laboratory” for studying how people behave and perform in high-pressure situations. Sports have traditionally been gender segregated, with separate tournaments and opportunities for men and women. As a result, gender differences in sports have sparked much attention, allowing us to investigate the physical, mental, and social elements that influence people’s athletic performance (Handelsman et al., 2018).

Previous research findings into gender disparities in resilience have been inconsistent and contradictory. A considerable amount of literature has found gender-based differences in response to setbacks. Weinberg and Jackson (1989) studied gender disparities in tennis players’ ability to win a match after losing the first set. Overall, males were more likely to come from behind than females among junior-aged players. Gill and Prowse (2014) find that women take discouraging news worse than men do. Similarly, some studies have found that women perform just as well as men in high-pressure scenarios such as teaching or professional tennis (Paserman, 2007; Lavy, 2012). However, Banko et al. (2016) analyzed tennis data to determine whether females react more negatively to setbacks than males. And they did not discover any difference in their reactions. Recent research conducted by Toma (2017) and Bucciol and Castagnetti (2020) reveals that men and women are equally prone to choking under pressure.

Some studies are dedicated to examining gender differences in competitiveness during the critical stages of competitions. Analyzing tennis data, the detailed point-by-point analysis reveals that, relative to men, women are substantially more likely to make unforced errors at crucial junctures of the match (Paserman, 2023). However, Cohen-Zada et al. (2017b) discovered that only men’s performance decreases following a loss in a bronze medal fight.

Other studies on gender differences in professional sports concentrated on financial incentives and their effects on performance (Maloney and McCormick, 2000; Deaner, 2006; Gilsdorf and Sukhatme, 2008; Lallemand et al., 2008; Frick, 2011). All studies show women succumbing to the pressure that comes with significant prizes at risk. Other research discovered that men professional golfers (PGA) performed better when their financial benefits increased (Ehrenberg and Bognanno, 1990a; Ehrenberg and Bognanno, 1990b). However, another study failed to duplicate the men’s results using the 1992 PGA records (Orszag, 1994), and overall results in this area of research are mixed.

On the other hand, in academic settings, other studies suggest that men outperform women under increased competition (Orszag, 1994; Azmat et al., 2016; Cai et al., 2019). Gneezy et al. (2003) find that the relative and absolute performance of males increases as competition intensifies. The performance of female students dominates that of male students in the less competitive exam, whereas the opposite holds in the more competitive exam. A similar picture emerges from the study by Morin (2015).

According to the above literature, women are less resilient to stress than men, except in the sport of tennis where this is controversial. We examine data from professional recurve archery players on the men’s and women’s tours to determine if women react worse to competitive failures or in the critical stage of competition. We aim to understand not only whether female and male contestants react differently to poor performance in the past but also to explore the potential psychological mechanisms, such as self-confidence and competitive anxiety, that may drive these differences. Our key findings are significant in understanding the impact of feedback on performance. Professional sports provide a fruitful setting for studying gender differences in decision-making and performance (Kahn, 2000; Paserman, 2007; Cohen-Zada et al., 2017a), as well as behavioral differences between men and women (Malueg and Yates, 2010; González-Díaz et al., 2012; Banko et al., 2016). Archery statistics contain three valuable attributes for examining gender disparities. Optimal shooting performance is typically achieved when arousal levels are low or moderate. Hence, it necessitates precise motor control, stability, and the capacity to avoid involuntary muscular contractions (Robazza et al., 1998). Such attributes of archery, which differentiate it from more intense sports such as judo or tennis, enable us to disregard the influence of physiological (testosterone) and physical factors on gender disparities. Furthermore, no bewildering impacts arise from collaborative gameplay, defensive tactics, or score-dependent strategies. Elite archers consistently prioritize attentiveness to each arrow and strive to achieve optimal accuracy by targeting the center. Ultimately, male and female athletes experience the same competitive settings. Both male and female individual matches follow a best-of-five format. Every shot is captured from a consistent distance, with brief time intervals separating them.

Our first objective in the current study is to identify cold-hand effects in archery and whether the magnitude of the cold-hand effect is significantly different between men and women. We specifically want to find out if the last arrow in each set performs worse after the previous worse performance. The concept of “cold-hand” sheds light on the hot-hand literature.

There is a lot of research supporting the hot-hand effect, but there is less research on the cold-hand effect (Arkes, 2010; Yaari and Eisenmann, 2011; Bocskocsky et al., 2014; Miller and Sanjurjo, 2015), with only limited literature supporting it (Arkes, 2016). Cold hand effect is a negative momentum, which predicts that failure increases the probability of subsequent failure. Following this, our primary aim is to examine whether these impacts vary by the athletes’ gender.

The second empirical goal of this paper is to determine whether the magnitude of the gender disparity differs according to the game’s critical stage, or the final arrow’s dynamic game state in a given set. For this reason, we categorize the competitive situation into four types. We speculate that under condition C1 (losing the current set will result in the loss of the entire game while winning the set will have no impact on the outcome of the game), the athletes experience the greatest pressure, and in such condition, they must win the next set to avoid elimination, encounter the adversity. We attempt to utilize the aforementioned indicators to discover the gender gap in the ability to bounce back from adversity.

## 2 Materials and methods

### 2.1 Recurve archery data

**Figure 1:**
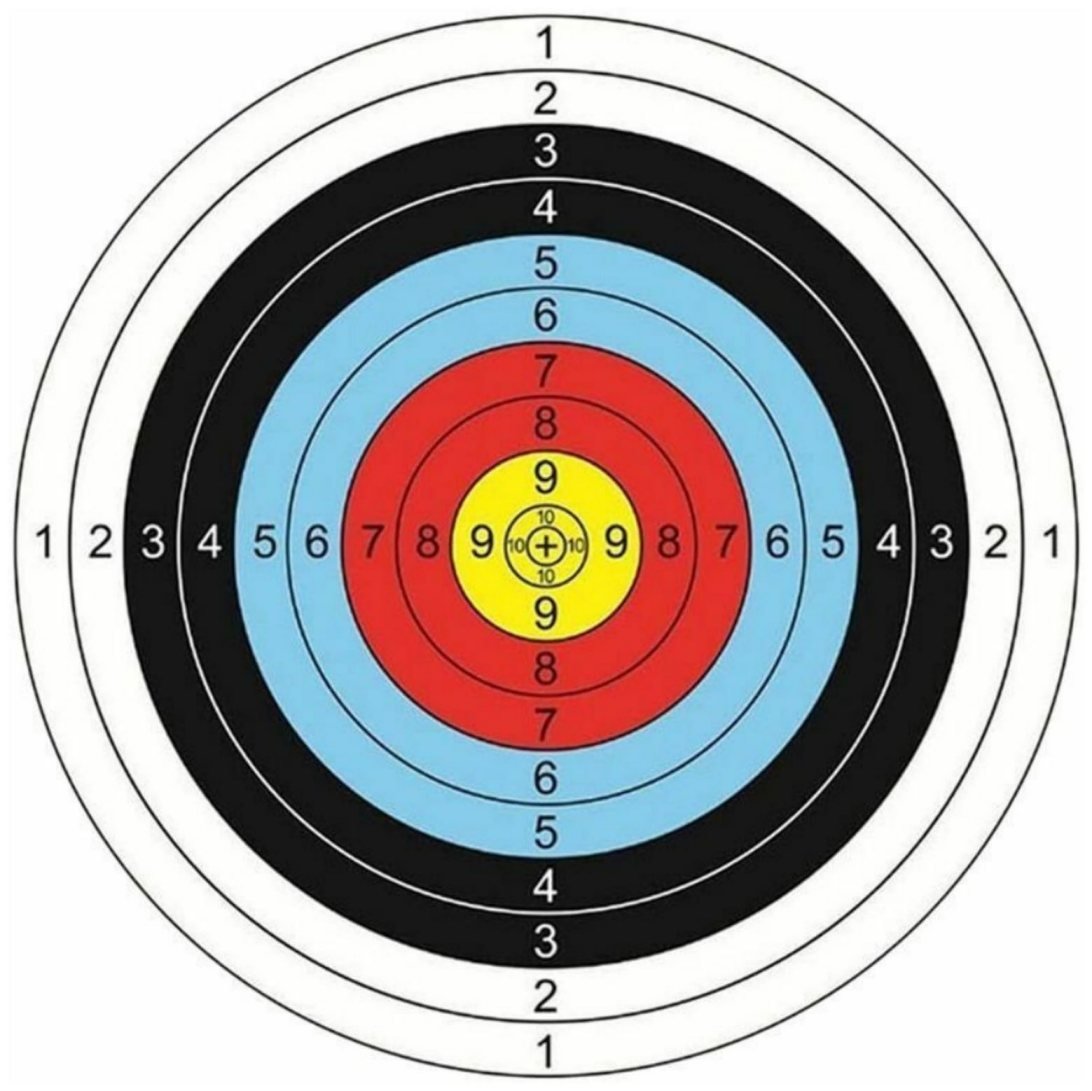
Recurve target face.

The target in recurve archery is 70 meters away. It has 10 concentric rings, with the center scoring 10 points and the outside circles scoring less (Figure 1). No points are awarded if an arrow misses the target completely.

Tournaments comprise two phases: the “ranking round” and the “elimination round.” During the ranking round, athletes shot a total of 72 arrows in order to determine their rankings. During the elimination rounds, athletes of comparable rankings compete against one another. The highest-ranked athlete competes against the lowest-ranked athlete; the second-highest-ranked athlete competes against the second-to-last competitor, and so on. Each match ends with the loser falling out and the winner advancing. This continues until just two competitors compete in the final. Semifinal losers compete for third place.

In a match, athletes compete in a best-of-five set format. Each set contains three arrows for each participant. The winner of a set receives two points, while a tie awards both competitors one point. The first player to reach six points wins. Each set begins with the higher-ranked athlete shooting first, and then the athlete with fewer points shoots first in the next set.

In current study, elite athletes are those who compete in international (London 2012, Rio 2016, Tokyo 2020 Olympics, Yankton 2021 Hyundai World Archery Championships) and continental championships (Antalya European Grand Prix 2021). Athletes who were unable to participate in the above events were excluded due to the unavailability of competition data. We have collected arrow-by-arrow data from various archery events such as the London 2012, Rio 2016, Tokyo 2020 Olympics, the Yankton 2021 Hyundai World Archery Championships, and the Antalya European Grand Prix 2021. This data was obtained from the official World Archery Federation website.^1^ For the London 2012 Olympics archery game, we obtained the data from the archived website.^2^ After collecting the data, we examined it thoroughly for 10 years from 2012 to 2021. We discovered 6,374 instances of three consecutive shots, totaling 19,122 shots. For each set of three shots, we recorded the player’s identity, the points achieved, the shooting order, whether the player participated in the Olympics or other games, and the set points in the game when the shots were taken.

We thoroughly examined the arrow-by-arrow data spanning 10 years from 2012 to 2021. In total, we discovered 6,374 instances of three consecutive shots, totaling 19,122 shots. For each set of three shots, we recorded the points achieved, the player’s identity, the shooting order, whether the player participated in the Olympics or other games, and the set points in the game when the shots were taken.

### 2.2 Variables

Our analysis is aimed at understanding whether the previous poor results or unfavorable game status affect subsequent players’ performance and whether this effect is heterogeneous according to gender. Our empirical strategy is quite simple: We first analyze the respective scores of archers in their third arrow in relation to the outcome of the previous two shootings. Specifically, we compared the points scored on the third shot under four scenarios: after two consecutive 10s (Momentum A, Hit-Hit), after a first shot of 10 and a second miss of 10s (Momentum B, Hit–Miss), after a first miss of 10 and a second shot of 10 points (Momentum C, Miss-Hit), and after two consecutive missing 10s (Momentum A, Miss–Miss). If a cold-hand effect exists, we expect to observe lower scores on the third shot following two consecutive 10s than following the other combinations. If an archer’s performance significantly decreases after missing 10 points compared to those hitting 10 points, we consider it to be the existence of a cold-hand effect (Table 1). To investigate whether men and women respond differently to the outcome of the first two arrows, we conducted a regression analysis. We used the performance of the third arrow as the dependent variable and the outcome of the previous two shootings (momentum type) as independent variables. The momentum type is crucial for understanding the cold-hand effect, with “Hit–Hit” scenarios potentially reinforcing performance, while “Miss–Miss” scenarios are expected to exacerbate performance declines. A deeper exploration of how these scenarios interact with gender could offer richer insights. We also included the gender of the player as an interaction term. Next, we performed the same analysis using game status as an additional independent variable. To account for differences in players’ abilities and heterogeneity, we used both players’ scores in ranking rounds.

**Table 1:** Definition and descriptive statistics of dependent and independent variables (*N* = 6,374).

Variable	Name	Type	Classification	Mean	Median	Std	Min	Max
Dependent variable	Arrow3	Continuous	Player’s points on their third shot	8.25	9	1.468	0	10
Independent variable	Momentum	Categorical	We then classify the momentum type into four categories: “A” (Hit–Hit, *n* = 827), “B” (Hit–Miss, *n* = 1753), “C” (Miss–Hit, *n* = 675), and “D” (Miss–Miss, *n* = 3,119)					
	Gender	Dummy	1 = male (*n* = 3,380), 0 = female (*n* = 2,994)					
	Status	Categorical	C0 (*n* = 4,108), C1 (*n* = 999), C2 (*n* = 268), C3 (*n* = 999)					
	Player heterogeneity	Continuous	=one archer’s ranking score/opponent’s ranking score	1.002	1	0.057	0.648	1.543
	Game type	Dummy	1 = Olympic games (*n* = 3,236), 0 = others (3138)					
	Order	Dummy	1 = second (*n* = 3,187), 0 = first (3187)					

“Hit” means hit the bullseye, “Miss” does not. “-” left is the first arrow score, right is the second arrow score. Numbers in parentheses represent sample sizes.

#### 2.2.1 Game status

The game status is separated into four categories: “C0” (winning or losing the current set has no impact on the outcome of the game), “C1” (losing the current set will result in the loss of the entire game while winning the set will have no impact on the outcome of the game), “C2” (winning the set wins the game, otherwise losing the game.), “C3” (winning the set will win the game; losing the set will not impact the game’s result). Status C0 occurs in a non-decisive set, hence we assume that the pressure it generates is the lowest. The other three states all occur in the decisive set, and we believe that their pressure is ranked from highest to lowest as C1, C2, C3. Similarly, losing the current set in state C1 and C2 means being eliminated, while in state C3, it does not lead to elimination. And winning the current set in state C2 means ultimate victory, while winning the current set in state C1 only means being spared from elimination.

#### 2.2.2 Player heterogeneity

This is based on the apparent fact that a player’s overall skill level influences their performance. Compared to bad shooters, proficient archers are more likely to shoot better on their third attempt. We used their ranking round score to estimate the strength ratio (score ratio = player’s ranking score/opponent’s ranking score) between the two players in a match, accounting for variations in athlete ability.

#### 2.2.3 Tournament type

We documented whether the match took place during the Olympics or the World Championship games to ascertain whether there was a competitive edge during the Olympics. The binary variable “Game Type” equals one for the Olympics (London 2012, Rio 2016, Tokyo 2020 Olympics) and zero for others (Yankton 2021 Hyundai World Archery Championships, Antalya European Grand Prix 2021).

#### 2.2.4 Shooting order

Individual competitions adopt alternating shooting; the higher-ranked archer shoots first in the first set, and the archer with lower set points shoots first in the next set. Thus, the dummy variable Order is one for shooting first and zero otherwise.

### 2.3 Statistical analysis

Since the dependent variable is a count of integers, we used a Poisson general linear model (GLM), which detected under-dispersion (Zuur et al., 2009). To correct for this, we used a quasi-GLM with the variance given by *φ* × *μ*, where μ is the mean and *φ* is the dispersion parameter estimated at 0.231. This adjustment means that all standard errors were multiplied by 0.481 (the square root of 0.231). It should be mentioned that under-dispersed count data can be handled using the quasi-Poisson approach (Hostetler et al., 2012; Otterbeck et al., 2019). While a negative binomial regression model could address potential overdispersion, the quasi-Poisson model was chosen due to its better fit for the specific data characteristics observed in this study.

For all GLM analyses conducted in this paper, the “Multicomp” package was used to conduct pairwise comparisons of means. The statistical software package R (R development core team 2018) was utilized for statistical analyses and graphing. All tests were two-tailed, and a *p*-value below 0.05 was considered significant.

Our final method is a Poisson regression-based test for the cold hand that examines how the performance of the last arrow per set depends on the performance of the previous two shots.

We also interact the player’s gender with performance on the previous two arrows and game status to test for gender differences in response to past performance and intermediate game status.

Our Poisson regression takes the following form:


$$\mathrm{Arrow}3_{i}\sim \mathrm{Possion}\left(\mu_{i}\right)\;\mathrm{and}\;E\left(Y_{i}\right)=\mu_{i}\;\mathrm{and}\;var\left(Y_{i}\right)=\varphi \times \mu_{i}$$

$$\begin{array}{l} \log\left(\mu_{i}\right)=\alpha +\beta_{0}\;{\mathrm{Momentum}}_{i}+\beta_{1}\;{\mathrm{Game Status}}_{i}+\\ \beta_{2}\;{\mathrm{Heterogeneity}}_{i}+\beta_{3}\;{\mathrm{Game Type}}_{i}+\beta_{4}\;{\mathrm{Order}}_{i}+\\ \beta_{5}\;{\mathrm{Gender}}_{i}+\beta_{6}\;{\mathrm{Momentum}}_{i}\times {\mathrm{Gender}}_{i}+\\ \beta_{7}\;{\mathrm{GameStatus}}_{i}\times {\mathrm{Gender}}_{i}+\varepsilon_{i} \end{array}$$

$\mathrm{Arrow}3_{i}$ , with the number of points on the last arrow per set i , is Poisson distributed with a mean of $\mu_{i}$ .

## 3 Results

### 3.1 Cold-hand effects

The statistically significant evidence of a cold-hand effect is presented in Table 2. This is supported by the negative and significant coefficients that are linked to previous worst performances [*t* = −12.862 e*^β^* = 0.878 (0.86–0.90), *p* < 0.001], specifically missing the bullseye twice in a row. It indicates significantly lower scores (12.2%) after two missing 10-point shots compared to the case after two 10-point shots.

**Table 2:** Quasi-Poisson regressions predicting average scores in the first and second versus third shots (*N* = 6,374).

Parameters	*β* (95% CI)	Stand error	*t*
Intercept	1.696 (1.619, 1.774)	0.04	42.857***
Gender (male vs. female)	0.014 (−0.009, 0.037)	0.012	1.215
**Momentum A#**			
Momentum B	−0.089 (−0.11, −0.067)	0.011	−8.112***
Momentum C	−0.075 (−0.1, −0.05)	0.013	−5.79***
Momentum D	−0.131 (−0.151, −0.111)	0.01	−12.862***
Player heterogeneity	0.41 (0.337, 0.483)	0.037	11.011***
Type (olympic vs. champion)	0.151 (0.143, 0.16)	0.004	35.279***
Order (second vs. first)	0 (−0.01, 0.01)	0.005	0.017
**Status: C0#**			
Status: C1	−0.017 (−0.035, 0)	0.009	−1.944
Status: C2	−0.02 (−0.049, 0.008)	0.015	−1.377
Status: C3	0.004 (−0.014, 0.021)	0.009	0.408
Gender × Momentum B	0.025 (−0.002, 0.052)	0.014	1.828
Gender × Momentum C	0.004 (−0.028, 0.036)	0.016	0.233
Gender ×Momentum D	0.035 (0.01, 0.06)	0.013	2.75**
Gender × C1	0.024 (0.001, 0.046)	0.011	2.075*
Gender × C2	0.004 (−0.036, 0.044)	0.02	0.212
Gender × C3	−0.002 (−0.024, 0.02)	0.011	−0.212
ϕ	0.21		
pseudo-*R*^2^	0.231		

***, **, and * denotes statistical significance at the 0.1, 1, and 5% levels, respectively. *β* denotes estimated coefficients. # denotes Reference categories. ϕ denotes dispersion parameters.

**Figure 2:**
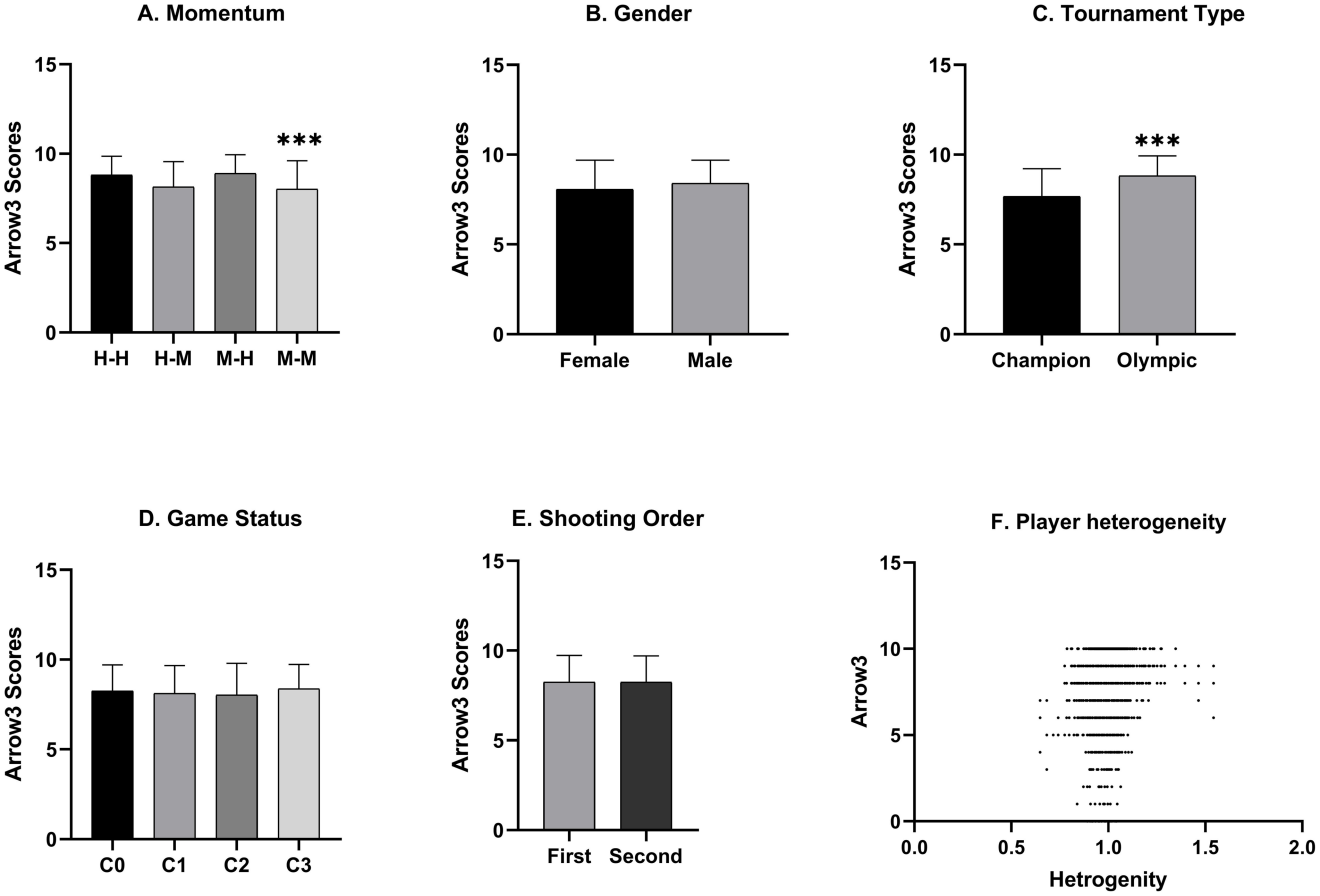
Graphical plots for simple main effects. **(A)** The third arrow scores versus momentum. **(B)** The third arrow scores versus sex. **(C)** The third arrow scores versus tournament. **(D)** The third arrow scores versus status. **(E)** The third arrow scores versus shooting order. **(F)** The third arrow scores versus player heterogeneity.

Moreover, the conditional score of the third shot after two missing 10-point shots is significantly higher than the average score following the other cases (*p* < 0.001, Figure 2A).

In conclusion, Table 2 shows that archers tend to shoot worse after missing the bullseye twice, regardless of the player’s gender, general skill, game status, shooting order, or game type. The cold-hand effect, while statistically significant, also presents practical implications for coaching strategies, suggesting that interventions should focus on enhancing resilience after consecutive poor performances.

### 3.2 Control variables

In Poisson regression, we see that the player’s general skill has a highly significant effect on their conditional score for the third shot (Figure 2F). This takes into account the size and statistical importance of heterogeneity. And this control variable is the most important thing in these regressions. Also, the “Game Type” binary variable shows a higher score on the third shot per set in Olympic games than in Championship games (Figure 2C). Both the player heterogeneity and game type effect are significant at the 0.0001 level.

Furthermore, when in game status C1 (losing the current set will result in the loss of the entire game while winning the set will have no impact on the outcome of the game), archers perform worse on the third arrow than when it is in game status C0 (winning or losing the current set has no impact on the outcome of the game, Figure 2D). However, none of these differences among statuses were statistically significant (Table 2). Similarly, the order of archery had no significant effect on the performance of the third arrow (*t* = 0.017, *p* = 0.986).

By including these variables in our study, we improve the reliability of our findings and reinforce the conclusion that the cold-hand phenomenon is present in repeated archery attempts.

### 3.3 Gender differences

**Figure 3:**
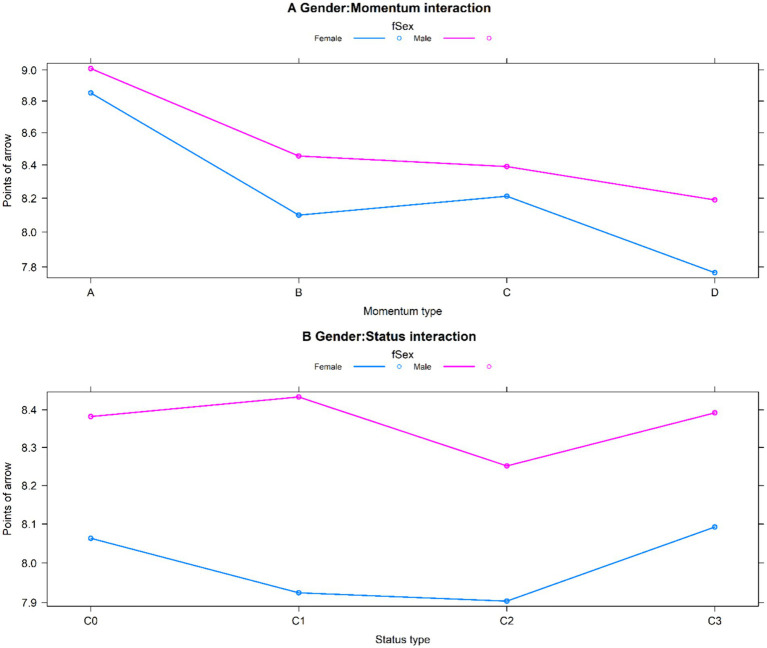
Graphical plots for interaction effects. **(A)** gender by momentum type interaction; **(B)** gender by status interaction.

Although male archers scored slightly higher than female archers on the last arrow of each set (Figure 2B), this difference was not significant (*t* = 1.214, *p* = 0.225). Table 2 shows that the coefficient (0.035) for the interaction between gender and the previous worst performance (missing bullseye twice) is positive. This information indicates that when archers get equal bad scores on the first two arrows, male athletes do much better on the third arrow than female athletes, with a 3.5% difference (*p* < 0.001, Figure 3A).

Another significant aspect of the interaction effect is between gender and game status. In a situation where losing can only be avoided by winning, the third arrow performance gap between male and female athletes significantly increased by 2.4% (Table 2). This suggests that the decline in the performance of female athletes on the third arrow is considerably higher than that of male athletes when on the verge of failure (game status C1, Figure 3B).

## 4 Discussion

This paper uses archery, an individual sport with an isolated single-sex structure, to verify potential gender differences in competitive behaviour. We find evidence supporting the existence of a negative momentum (cold-hand) effect—that is, a poor performance on the previous two shots leads to a worse performance on the final shot. Such supportive evidence in favour of the “failure breeds failure” indicates the presence of cold-hand effect.

Our study enables us to conclude that gender differences are more pronounced in reaction to a negative result. In a situation where losing can only be avoided by winning or facing poor previous performance, female athletes have 2.4 and 3.6% lower performance than their male counterparts. The above results indicate a gender difference in the ability to handle pressure between men and women in elite recurve archery. Similar findings on choking under pressure in archery have been discussed by Diotaiuti et al. (2021), who explored the psychological factors contributing to performance declines in high-stakes situations.

Consequently, our findings complement those of Banko et al. (2016), who found that women perform considerably worse when trailing by a substantial margin. This finding is consistent with that of De Paola and Scoppa (2017), who indicate that women can handle pressure just as well as men, as long as they are not lagging.

Regarding the gender difference in handling pressure from near setbacks, the results are inconsistent. Lackner and Weichselbaumer (2023) find that women perform considerably worse after near setbacks in tennis, while Banko et al. (2016) suggest that women are not more prone to losing due to setbacks. These findings align with recent research by Diotaiuti et al. (2021) on choking episodes in archery, which also highlight the importance of psychological resilience in maintaining performance under pressure.

We attempt to explain the reasons behind the results above from both psychological and physiological perspectives. From a psychological perspective, the following four types of gender differences can explain the above-mentioned gender differences.

### 4.1 Gender differences in self-confidence

Published reports suggest that men generally have higher self-confidence than females in competitive situations (Lirgg, 1991; O’Connor et al., 2020). Research has demonstrated that receiving negative feedback can diminish self-assurance, especially in women (Roberts and Nolen-Hoeksema, 1989), perhaps leading to decreased performance (Woodman and Hardy, 2003). Lirgg (1991) emphasized the possibility that males tend to overestimate their performance, whereas females may underestimate their performance, which can lead to differences in confidence ratings (Lirgg, 1991).

Undoubtedly, self-confidence must play a role in archers’ ability to bounce back from adversity. Self-confidence will impact archers’ subsequent motivation and performance after an abysmal performance or facing near failure. According to prior theory and research, males may be more resilient to such a loss due to a higher level of self-confidence. On the other hand, because females’ self-confidence may be more sensitive to feedback, a setback may be more likely to reduce females’ self-confidence and interfere with subsequent performance.

### 4.2 Gender differences in competitive anxiety

Male athletes typically display lower levels of anxiety than female athletes (Krane and Williams, 1994; Correia and Rosado, 2019). Female athletes exhibited higher levels of competitive trait anxiety (Kristjánsdóttir et al., 2018) and higher levels of worries (O’Donoghue and Neil, 2015).

The intensity and direction of competitive trait anxiety were investigated by Perry and Williams (1998) based on gender disparities (Perry and Williams, 1998). While cognitive anxiety levels were not different between males and females, females were more likely to have a debilitating interpretation of it.

Females reported that cognitive anxiety was a hindrance to tennis performance. In addition, males more often interpreted somatic and cognitive anxiety as facilitative to competitive performance. The female archers exhibited higher levels of felt arousal and cognitive anxiety than the male archers.

Gender differences in levels of competitive trait anxiety might contribute to the understanding of potential gender differences in archers’ ability to overcome a disadvantageous situation.

### 4.3 Gender differences in athletic coping

Studies have shown that males tend to employ problem-focused coping strategies more frequently, while females rely more on emotion-focused coping strategies (Tamres et al., 2002; Hammermeister and Burton, 2004; Anshel et al., 2009). Psychological factors of competitive anxiety are related to coping strategies (Hoar et al., 2010). Active coping, planning, effort, and suppression of competing activities are examples of problem-focused coping methods that have a positive correlation with positive affect (Watson et al., 1988), which is a reflection of enjoyable engagement. Positive affect was also found to be inversely correlated with behavioural disengagement and wishful thinking (Gaudreau et al., 2002).

These findings have implications for archers’ responses to frustration in a match. If females are more likely to resign after a failure and males are more likely to engage in problem-focused strategies, males are more equipped to overcome a loss.

### 4.4 Gender differences in competitiveness

Extensive literature confirms the presence of a significant and enduring disparity between genders in terms of competition (Frick, 2011; Wozniak, 2012; Deaner et al., 2016; Birk et al., 2019). Trait competitiveness measures have shown that males are more competitive than females. Studies showing females as more competitive have had small samples of high-level athletes, which are not typical of females. In contrast, the male samples were more prominent and more representative. Although the results are inconsistent, there seems to be reasonable support for this gender difference. A high level of competitiveness would likely contribute to an archer’s motivation to overcome a loss.

Summing up, differences exist between the two gender groups in a variety of fields such as confidence, anxiety, coping strategy, and competitiveness. Such variation may explain the causes of sex differences in response to failures.

From a physiological perspective, poor performance in recurve archery was correlated with high real-time heart rate (Lu and Zhong, 2023). Some heart rate variability (HRV) studies have indicated that females may exhibit a higher overall complexity of heart rate dynamics than males (Ryan et al., 1994). Women show a greater mean heart rate (Koenig and Thayer, 2016). It should be mentioned that a higher heart rate—which indicates an increase in psychological stress—is associated with lower scores. Archery is a sport that requires fine movement control, and postural stability is considered an important variable in achieving high performance (Sarro et al., 2021). An increased heart rate can accelerate the imbalance of the human body posture and reduce the ability of people to maintain body balance posture. Subsequently, it affects shooting performance.

Furthermore, the gender-specific autonomic differences may contribute to the weaker resilience to setbacks in females. For example, during adolescence, girls are 3 times more likely to develop post-traumatic stress disorder (PTSD) than boys (McLaughlin et al., 2013). Sex or gender differences in cognitive styles contribute to resilience for post-traumatic stress disorder (PTSD) and other mood disorders.

### 4.5 Limitation

The absence of qualitative data on psychological factors and athletes’ backgrounds limits the depth of the conclusions that can be drawn. Unfortunately, the constraints of our data prevent a thorough investigation into the disparities in how men and women respond to failure across various sports, the labour market, and marital outcomes. The results may not be fully applicable to other sports or competitive contexts where the dynamics of pressure and feedback differ significantly. Additionally, the dataset lacks detailed information on psychological interventions or external support factors, which could play a significant role in shaping athletes’ responses to pressure. Furthermore, our analysis is limited in its capacity to elucidate the underlying causes for the observed gender differences in the archery context. The observed gender differences in response to consecutive poor performances may be influenced by other factors such as experience level, access to psychological support, and prior exposure to high-pressure situations, which should be considered in future research. It is recommended that future studies, equipped with more extensive data, delve deeper into these issues to provide a more comprehensive understanding of high-level performance under stress.

## 5 Conclusion

We utilize large-scale data from elite recurve archery to investigate gender differences in performance feedback. The detailed arrow-by-arrow analysis reveals that, relative to men, women are substantially more likely to react negatively to negative feedback. We have found that women tend to experience a stronger discouragement effect than men when in a situation where losing can only be avoided by winning or facing recent poor performance. The insights gained from this study may be of assistance to investigate the gender gap in the ability to handle pressure.

## 6 Practical applications

Our findings can help us understand why gender differences occur in various situations where individuals compete one after another and receive feedback on their interim performance. Furthermore, future investigations should conduct more comprehensive analyses using simulations to enhance the statistical robustness of our discoveries and gain deeper insights into the presence of the hot hand or momentum phenomenon. For instance, in a professional context, training programs could be designed to enhance resilience in female employees, drawing parallels with strategies used to bolster performance in high-pressure sports like archery.

## Data Availability

The datasets generated for this study are available on request to the corresponding author.

## Footnotes

^1^ https://worldarchery.sport/events/results

^2^ https://webarchive.nationalarchives.gov.uk/ukgwa/20130301124206/http://www.london2012.com/archery/event/men-individual/
